# Roles of arabidopsis WRKY18, WRKY40 and WRKY60 transcription factors in plant responses to abscisic acid and abiotic stress

**DOI:** 10.1186/1471-2229-10-281

**Published:** 2010-12-19

**Authors:** Han Chen, Zhibing Lai, Junwei Shi, Yong Xiao, Zhixiang Chen, Xinping Xu

**Affiliations:** 1 State Key Laboratory of Biocontrol and Key Laboratory of Gene Engineering of the Ministry of Education, School of Life Sciences, Sun Yat-sen University, Guangzhou 510275, China; 2 Department of Botany and Plant Pathology, Purdue University, West Lafayette, IN 47907-2054, USA

## Abstract

**Background:**

WRKY transcription factors are involved in plant responses to both biotic and abiotic stresses. Arabidopsis WRKY18, WRKY40, and WRKY60 transcription factors interact both physically and functionally in plant defense responses. However, their role in plant abiotic stress response has not been directly analyzed.

**Results:**

We report that the three WRKYs are involved in plant responses to abscisic acid (ABA) and abiotic stress. Through analysis of single, double, and triple mutants and overexpression lines for the WRKY genes, we have shown that *WRKY18 *and *WRKY60 *have a positive effect on plant ABA sensitivity for inhibition of seed germination and root growth. The same two WRKY genes also enhance plant sensitivity to salt and osmotic stress. *WRKY40*, on the other hand, antagonizes *WRKY18 *and *WRKY60 *in the effect on plant sensitivity to ABA and abiotic stress in germination and growth assays. Both *WRKY18 *and *WRKY40 *are rapidly induced by ABA, while induction of *WRKY60 *by ABA is delayed. ABA-inducible expression of *WRKY60 *is almost completely abolished in the *wrky18 *and *wrky40 *mutants. WRKY18 and WRKY40 recognize a cluster of W-box sequences in the *WRKY60 *promoter and activate WRKY60 expression in protoplasts. Thus, *WRKY60 *might be a direct target gene of WRKY18 and WRKY40 in ABA signaling. Using a stable transgenic reporter/effector system, we have shown that both WRKY18 and WRKY60 act as weak transcriptional activators while WRKY40 is a transcriptional repressor in plant cells.

**Conclusions:**

We propose that the three related WRKY transcription factors form a highly interacting regulatory network that modulates gene expression in both plant defense and stress responses by acting as either transcription activator or repressor.

## Background

Plants are constantly exposed to a variety of biotic and abiotic stresses and have evolved intricate mechanisms to sense and respond to the adverse conditions. Phytohormones such as salicylic acid (SA), ethylene (ET), jasmonic acid (JA) and abscisic acid (ABA) play important roles in the regulation of plant responses to the adverse environmental conditions. In Arabidopsis, mutants deficient in SA biosynthesis (e.g. *sid2*) or signalling (e.g. *npr1*) exhibit enhanced susceptibility to biotrophic pathogens, which parasitize on plant living tissue [1,2]. ET- and JA-mediated signaling pathways, on the other hand, often mediate plant defense against necrotrophic pathogens that promote host cell death at early stages of infection [3]. ABA is extensively involved in plant responses to abiotic stresses including drought, extreme temperatures and osmotic stress [4,5]. ABA also plays a regulatory role in important plant growth and developmental processes including seed development, dormancy, germination and stomatal movement. Recent studies have reported crosstalk of signaling pathways regulated by these signal molecules that contributes to either antagonistic or synergistic interactions between abiotic and biotic interactions [6,7].

A large body of evidence indicates that plant WRKY DNA-binding transcription factors play important role in plant defense responses. In Arabidopsis, a majority of its WRKY genes are induced by pathogen infection or SA treatment [8]. A large number of plant defense or defense related genes including pathogenesis-related (*PR*) genes and the regulatory *NPR1 *gene contain W box sequences in their promoters that are recognized by WRKY proteins [9]. A number of studies have shown that these W-box sequences are necessary for the inducible expression of these defense genes. Mutant analyses in Arabidopsis have revealed direct links between specific WRKY proteins and complex plant defense responses. Mutations of *WRKY70 *enhance plant susceptibility to both biotrophic and necrotrophic pathogens including *Erwinia carotovora*, *Hyaloperonospora parasitica*, *Erysiphe cichoracearum *and *Botrytis cinerea *[10-12]. *Disruption *of *WRKY33 *results in enhanced susceptibility to necrotrophic fungal pathogens and impaired expression of JA/ET-regulated defense genes [13]. Mutations of other WRKY genes including *WRKY7*, *WRKY11*, *WRKY17*, *WRKY48*, *WRKY38 *and *WRKY62*, on the other hand, enhance basal plant resistance to virulent *P. syringae *strains, suggesting that they function as negative regulators of plant basal defense [14-17].

There is also evidence that WRKY transcription factors are involved in plant responses to abiotic stresses. Microarray experiments have identified WRKY genes that are induced by various abiotic stresses. In Arabidopsis, for example, WRKY genes were among several families of transcription factor genes that are induced by drought, cold or high-salinity stress [18-20]. The barley *Hv-WRKY38 *gene is rapidly and transiently induced during exposure to low non-freezing temperature in ABA-independent manner and exhibits continuous induction during dehydration and freezing treatment [21]. In tobacco, a WRKY transcription factor is specifically induced during a combination of drought and heat shock [22]. Regulated expression of WRKY genes during plant stress responses provides circumstantial evidence that implicates WRKY proteins in plant responses to abiotic stress. In Creosote bush (*Larrea tridentate*) that thrives in vast arid areas of North American, a WRKY protein (LtWRKY21) is able to activate the promoter of an ABA-inducible gene, *HVA22*, in a dosage-dependent manner [23]. A number of rice WRKY proteins regulate positively or negatively ABA signalling in aleurone cells [23,24]. Overexpression of soybean *GmWRKY13*, *GmWRKY21 *and *GmWRKY54 *conferred differential tolerance to abiotic stresses in transgenic Arabidopsis plants [25]. However, stable or transient overexpression of a gene in transgenic plants can often lead to pleiotropic phenotypes that may or may not reflect the true biological functions of the gene. Very recently, Jiang and Yu [26] have reported that Arabidopsis *wrky2 *knockout mutants are hypersensitive to ABA responses during seed germination and postgermination early growth, suggesting an important role of the stress-regulated WRKY gene in plant stress responses.

Arabidopsis *WRKY18*, *WRKY40 *and *WRKY60 *are pathogen-induced and encode three structurally related WRKY proteins [27]. We have previously shown that WRKY18, WRKY40 and WRKY60 interact physically with themselves and with each other through a leucine-zipper motif at their N-terminus [27]. Analysis with both knockout alleles and overexpresison lines indicated that the three pathogen-induced WRKY transcription factors have a partially redundant negative effect on SA-mediated defense but exerted a positive role in JA-mediated defense. [27]. Likewise, ABA plays a complex role in plant defense response. In Arabidopsis, ABA counteracts SA-dependent defense against the hemibitrophic bacterial pathogen *Pseudomonas syringae *[7], but is a signal required for resistance to the necrotrophic pathogens *Pythium irregulare *and *Alternaria brassicicola *[28]. In the present study, we report that Arabidopsis WRKY18, WRKY40 and WRKY60 proteins indeed function in a complex pattern in plant responses to ABA and abiotic stresses. The complex roles of the three WRKY transcription factors in plant biotic and abiotic stress responses are consistent with the complex nature of their expression, transcription-regulating activities and physical interactions.

## Results

### Altered ABA Sensitivity of Mutants and Overexpression Plants

**Figure 1:**
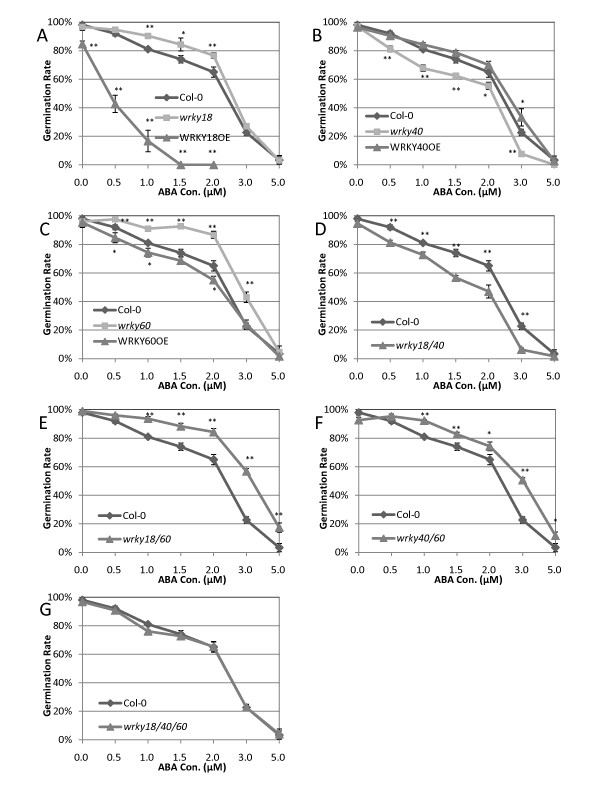
**Altered germination rates under exogenous ABA treatment**. Seeds of wild type, mutants and overexpression lines were sown on 1/2 MS media containing indicated concentrations of ABA. Seedlings with green cotyledons were considered as germinated. Germination rates were determined 120 hours after sowing. The means and standard errors were calculated from three independent experiments. (Asterisks: p-value < 0.05; Double Asterisks: p-value < 0.01).

To determine their possible roles in plant ABA response, we first performed germination experiments to analyze the ABA sensitivity of previously characterized knockout mutants and overexpression lines for *WRKY18*, *WRKY40 *and *WRKY60 *(Figure 1; Additional file 1). In the absence of ABA, 100% of wild-type seeds and more than 85% of *WRKY18*-overexpressing plants germinated (Figure 1A). In the presence of 0.5 and 1.0 μM ABA, however, the germination rates of *WRKY18*-overexpressing plants were reduced to 50% and 20% of those of wild type, respectively (Figure 1A). At 1.5 μM ABA, germination of *WRKY18*-overexpression plants was completely inhibited while almost 80% of wild-type seeds still germinated (Figure 1A). Thus, overexpression of *WRKY18 *enhanced seed sensitivity to ABA in germination assays. Disruption of *WRKY18*, on the other hand, significantly reduced plant sensitivity to ABA as indicated by an approximate 15% increase in the germination rates of the *wrky18 *mutant at 1.0, 1.5 and 2.0 μM ABA over those of wild-type plants (Figure 1A). Thus disruption of *WRKY18 *reduced seed sensitivity to ABA in germination assays. Similar results were observed for *WRKY60 *from the germination experiments. In the absence of ABA, the germination rates of both the knockout mutant and overexpression line for *WRKY60 *were similar to those of wild type (Figure 1C). When ABA was added to the medium, germination of the *wrky60 *mutant was less inhibited than that of wild type. For example, when ABA concentration was increased from 0 to 2 μM, there was only about 10% reduction in germination rate of the *wrky60 *mutant compared to more than 40% reduction of wild type (Figure 1C). Furthermore, overexpression of *WRKY60 *enhanced plant ABA sensitivity as indicated by significantly increase in inhibition of germination in the overexpression line relative to that of wild type (Figure 1C). Increased inhibition of germination in the *WRKY60*-overexpressing lines, however, was much less than that in the *WRKY18*-overexpressing line (Figure 1A, C). By contrast, the *wrky40 *knockout mutant was more sensitive and the overexpression line was less sensitive than wild type to the inhibitory effect of ABA on germination (Figure 1B).

We have previously shown that structurally related WRKY18, WRKY40 and WRKY60 interact both physically and functionally in the regulation of plant basal defense [27]. To determine possible functional interactions among the three WRKY proteins, we compared the ABA sensitivity of their double and triple knockout mutants (Figure 1D, E, G and 1F; Additional file 1). Germination rates of the *wrky18 wrky60 *double mutant at relatively low ABA concentrations (< 2 μM) were higher than those of wild type and were similar to those of the *wrky60 *single mutant (Figure 1E). At higher ABA concentrations (3 and 5 μM), however, the germination rates of the double mutant were 10-15% higher than those of the *wrky60 *single mutant (Figure 1E). Thus, WRKY18 and WRKY60 act additively in enhancing seed sensitivity to ABA in germination assays. The germination rates of the *wrky18 wrky40 *double mutant at various ABA concentrations were substantially lower than those of wild type and the *wrky40 *single mutant (Figure 1D). Interestingly, the germination rates of the *wrky40 wrky60 *double mutant were significantly higher than those of wild type. However, at certain ABA concentrations (e.g. 1.5 and 2.0 μM) the *wrky40 wrky60 *double mutant didn't germinate as well as the *wrky60 *single mutant (Figure 1). There was no significant difference between wild type and the *wrky18 wrky40 wrky60 *triple mutant in germination at the various ABA concentrations tested (Figure 1).

**Figure 2:**
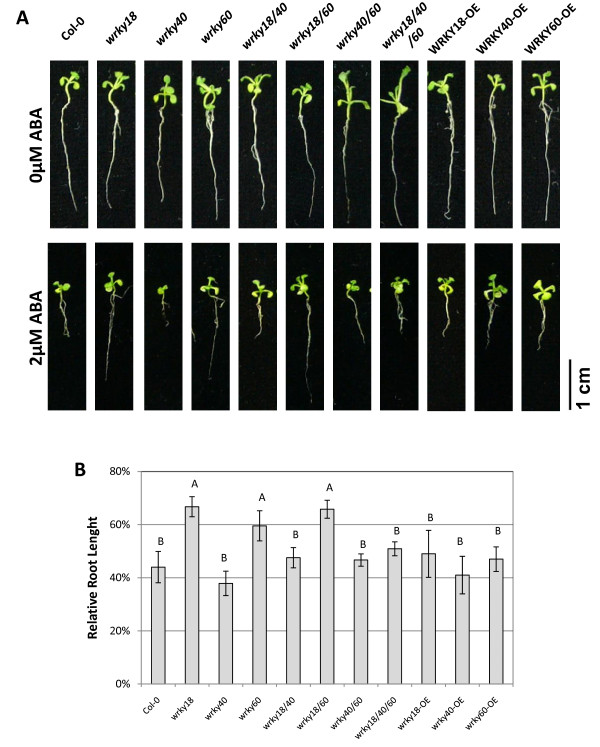
**Altered root elongation under exogenous ABA treatment**. Seeds of wild type and mutants were grown on 1/2 MS media for four days and then were transferred to MS agar media containing 0 or 2 μM ABA. The picture was taken and the root length was determined at the 7th day after the transfer. The relative root length was the ratio of average root length of seedlings in 2 μM ABA medium to those in 0 μM ABA medium. Standard errors were calculated from three independent experiments, every of which employed more than 25 seedlings of each genotype. Groupings were based on Student-Newman-Keuls Test, a = 0.05.

We also compared the loss-of-function mutants for ABA-inhibited root growth. When compared with wild type, these mutants had similar root elongation in the absence of ABA (Figure 2A). In the presence of 2 μM ABA, root elongation of the *wrky18 *and *wrky60 *single mutants and the *wrky18 wrky60 *double mutant was less inhibited while the *wrky40 *mutant was slightly but not statistically significantly more inhibited than that of wild type (Figure 2). Root elongation of *wrky18 wrky40*, *wrky40 wrky60 *double mutants and *wrky18*, *wrky40 wrky60 *triple mutant was similar to that of wild type (Figure 2).

### Altered tolerance of mutants and overexpression plants to abiotic stress

**Figure 3:**
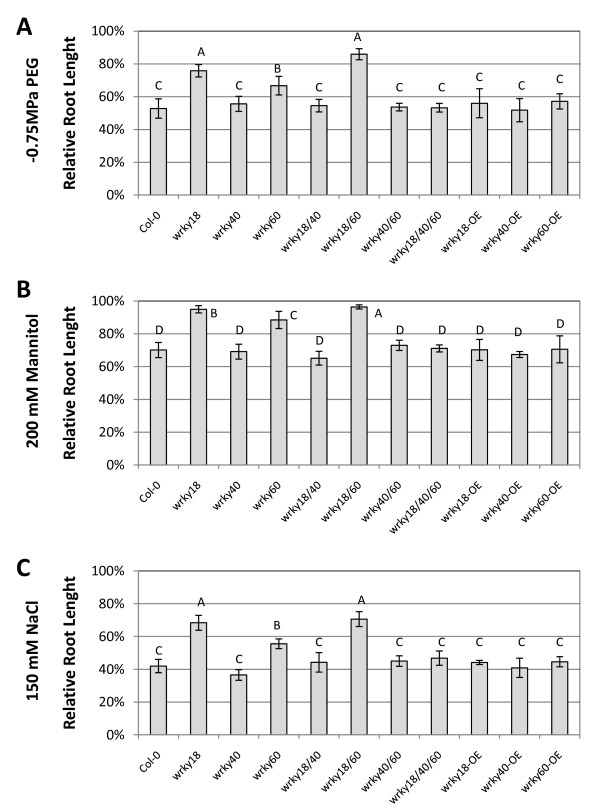
**Altered stress tolerance of the WRKY mutants**. Seeds of wild type and mutants were grown on 1/2 MS media for four days and then were transferred to MS agar media without or with -0.75 MPa PEG, 200 mM mannitol or 150 mM NaCl. The picture was taken and the root length was determined at the 7th day after the transfer. The average root length of each genotype in MS medium and their standard errors were calculated from three independent experiments, every of each employed more than 25 seedlings per genotype. Relative root length was the ratio of average root lengths of seedlings in medium with 200 mM mannitol, -0.75 MPa PEG or 150 mM NaCl to those in MS medium. The standard errors were calculated from three independent experiments, every of each employed more than 25 seedlings per genotype. Groupings were based on Student-Newman-Keuls Test, a = 005.

ABA is involved in plant responses to ionic and osmotic stresses. Since the *wrky18*, *wrky40 *and *wrky60 *mutants exhibited altered sensitivity to ABA in germination assays, we examined root growth of these mutants in growth media containing -0.75 MPa PEG, 200 mM mannitol or 150 mM NaCl. In the normal growth media, root elongations of all the mutants were similar to that of wild type (Figure 2). After transfer to the growth media containing PEG, mannitol or NaCl, the *wrky18*, *wrky60 *single mutants and *wrky18 wrky60 *double mutant was less sensitive than wild type to the osmotic and salt stress conditions (Figure 3; Additional file 2). Root elongation of the *wrky18 wrky40 *and *wrky40 wrky60 *double mutants and *wrky18 wrky40 wrky60 *triple mutant was similar to that of wild type under the osmotic and salt stress conditions (Figure 3; Additional file 2).

### Induced expression by ABA and abiotic stress

**Figure 4:**
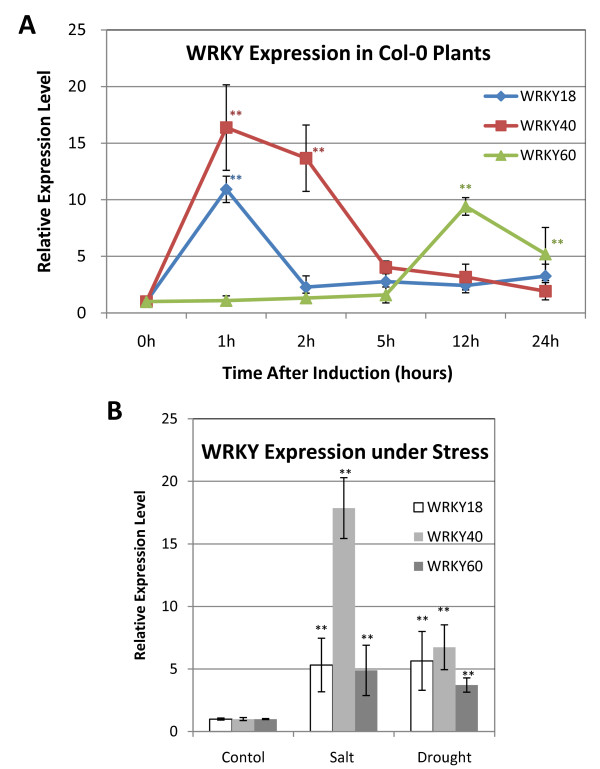
**Induced expression of WRKY genes by ABA and abiotic stresses**. A. Three-weeks-old wild-type plants were sprayed with water (Mock) or 5 μM ABA. Leaves from four treated plants were harvested at indicated time after the treatment for isolation of total RNA and analysis of transcripts using qRT-PCR. Expression level was defined as the ratio of qRT-PCR result of treated sample to its respective mock. The means and standard errors were calculated from three independent experiments. Asterisks mark statistically significant differences of expression level between ABA-treated-leaves harvested immediately and after indicated time. (Asterisks: p-value < 0.05; Double Asterisks: p-value < 0.01; by Student-Newman-Keuls Test). B. One-week-old wild-type seedlings were transferred to1/2 MS media without or with 150 mM NaCl or -0.75 MPa PEG. The seedlings were collected 24 hours after the transfer for total RNA isolation and analysis of transcripts using qRT-PCR. The means and standard errors were calculated from three independent experiments, all of which included no less than 20 seedlings per sample. Asterisks mark statistically significant differences of expression level between genotypically identical seedlings with or without indicated treatment. (Asterisks: p-value < 0.05; Double Asterisks: p-value < 0.01; by Student-Newman-Keuls Test).

*WRKY18, WRKY40 and WRKY60 *are induced in *Arabidopsis *plants upon infection by pathogen infection and SA [27]. Because of their role in plant response to ABA and abiotic stresses, we performed quantitative RT-PCR to analyze the effects of ABA and abiotic stresses on expression of these three WRKY genes. For determining ABA-regulated expression, we spraying three-week-old plants with 5 μM ABA and examined the transcript levels of the WRKY genes at 0 to 24 hours after the treatment. As shown in Figure 4A, the levels of *WRKY18 *and *WRKY40 *transcripts increased by about 10 and 16 fold during the first hour after ABA treatment, respectively. After 12 hours of ABA treatment, however, the transcript levels for both *WRKY18 *and *WRKY40 *were back to basal levels (Figure 4A), indicating that induction of the two WRKY genes by ABA was transient. By contrast, no significant increase in the transcript level of *WRKY60 *was observed after the first hour of ABA treatment. By 12 hours after the ABA treatment, the transcript level of WRKY60 was increased by about 10 fold above those of control plants (Figure 4A). The elevated levels of *WRKY60 *transcripts were still substantial even at 24 hour after the ABA treatment (Figure 4A). Thus, induction of *WRKY60 *by ABA was delayed but prolonged when compared to that of *WRKY18 *and *WRKY40*.

We also analyzed responses of the three WRKY genes to salt and drought(PEG) treatments. Wild-type seedlings (7 days old) were transferred to a MS growth medium with or without 150 mM NaCl or 250 g/l PEG and the seedlings were harvested 24 hours later for isolation of total RNA and qRT-PCR analysis. As shown in Figure 4B, the transcript levels for *WRKY18*, *WRKY40 *and *WRKY60 *were elevated by the NaCl treatment 6.5, 18.7 and 4.9 fold, respectively. After PEG treatment, the three WRKY genes were also induced 4 to 7 fold (Figure 4B). These results indicated that the three WRKY genes were also responsive to abiotic stresses. Induced expression of the WRKY genes by ABA and abiotic stresses have also been observed from previously reported microarray analysis [29,30].

**Figure 5:**
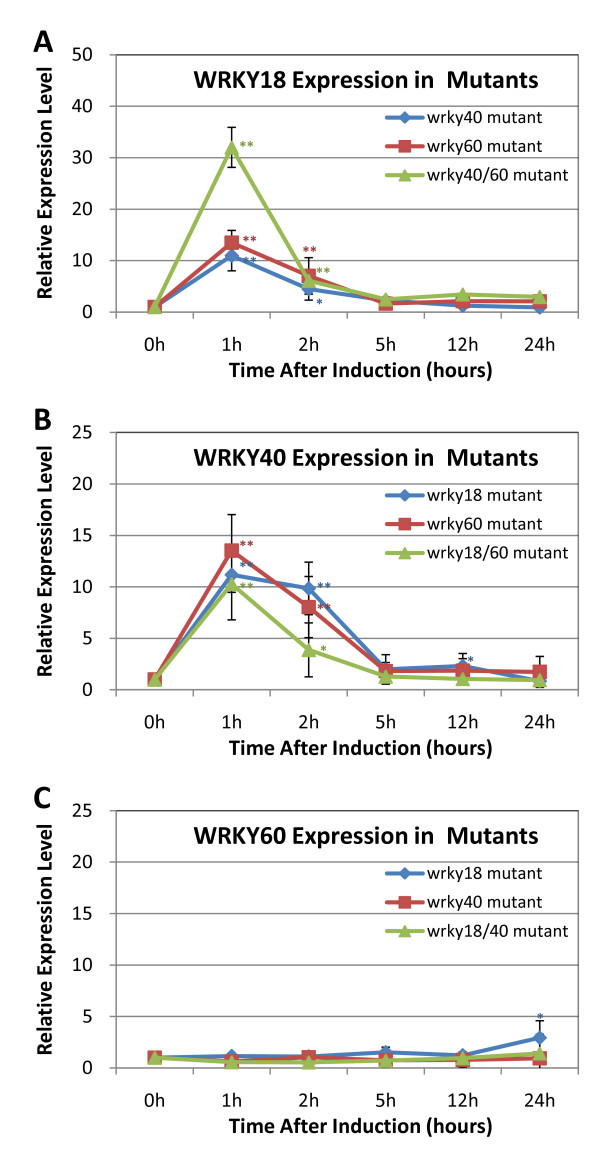
**WRKY18- and WRKY40-dependency of ABA-induced expression of WRKY60**. Three-weeks-old wild-type and mutant plants were sprayed with water (Mock) or 5 μM ABA. Leaves from four treated plants were harvested at indicated times after the treatment for isolation of total RNA and analysis of WRKY18 (A), WRKY40 (B) and WRKY60 (C) transcripts using qRT-PCR. Expression level was defined as the ratio of qRT-PCR result of treated sample to its respective mock. The means and standard errors were calculated from three independent experiments. Asterisks mark statistically significant differences of expression level between ABA-treated-leaves harvested immediately and after indicated time. (Asterisks: p-value < 0.05; Double Asterisks: p-value < 0.01; by Student-Newman-Keuls Test).

We have previously shown that pathogen-regulated WRKY genes are rich in W boxes in their promoters, suggesting that defense-regulated expression of WRKY genes involve extensive transcriptional activation or repression by its own members of the transcription factor family [8]. To examine possible mutual regulation among the three WRKY genes, we compared wild type and knockout mutants for ABA-regulated expression of the three WRKY genes. As described earlier, *WRKY18 *was rapidly and transiently induced by ABA in wild-type plants. A similarly rapid and transient induction of WRKY18 was observed in the *wrky40 *and *wrky60 *single mutants (Figure 5A). In the *wrky40 wrky60 *double mutant, induction of *WRKY18 *by ABA was also rapid and transient but the magnitude of induction was 2 -3 times higher than those of wild type and their parental single mutants (Figure 5A). Thus, WRKY40 and WRKY60 appear to play cooperatively a negative role in the induction of *WRKY18*. The levels *WRKY40 *transcripts also peaked at 1 hour after ABA treatment as observed for *WRKY18 *but the decline of WRKY transcripts after the first hour was somewhat slower than that of WRKY18 (Figure 5B). In addition, ABA induction of *WRKY40 *was slightly reduced in the *wrky18 *and *wrky60 *mutants (Figure 5B). Thus, WRKY18 and WRKY60 modulate positively induced expression of *WRKY40 *by ABA.

Induction of *WRKY60 *by ABA was relatively slow when compared to that of *WRKY18 *and *WRKY40 *(Figure 4A). In wild type, no significant induction of *WRKY60 *transcripts was observed during the first five hours after ABA treatment. However, *WRKY60 *transcripts increased about 10 fold by 12 hours after the treatment and then declined gradually during the remaining period of the experiments (Figure 4A). In the *wrky18 *mutant, the induction of *WRKY60 *was drastically reduced, with only a small increase observed after 24 hours of treatment (Figure 5C). In the *wrky40 *single mutant and *wrky18 wrky40 *double mutant, ABA induction of *WRKY60 *was completely abolished (Figure 5C). Thus both WRKY18 and WRKY40 are necessary for ABA-induced *WRKY60 *expression.

### Recognition of WRKY60 promoter by WRKY18 and WRKY40

**Figure 6:**
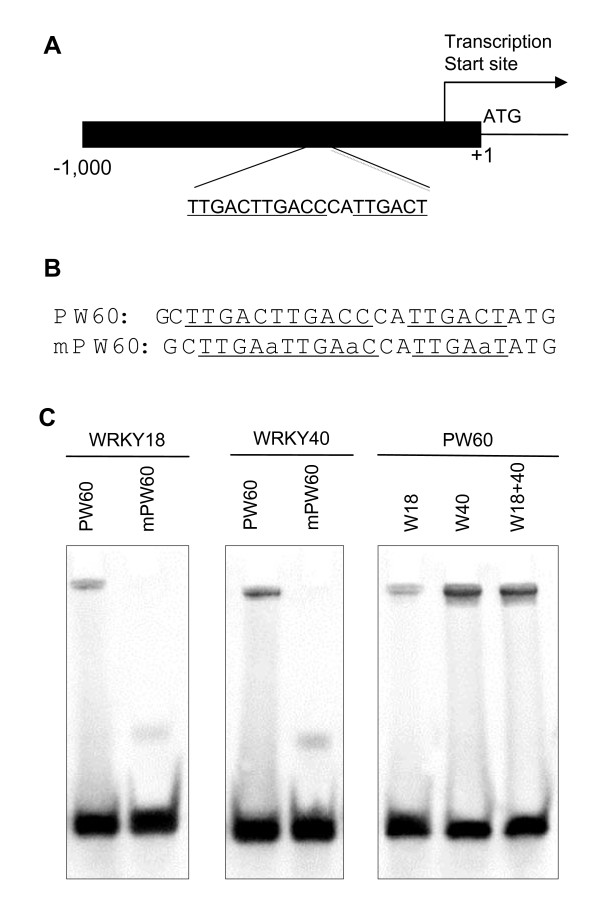
**Recognition of the WRKY60 promoter by WRKY18 and WRKY40**. A. Diagram of the WRKY60 gene, including the 1 kb upstream promoter that contains a cluster of three W-box sequences between -791 and -773 relative to the translation start codon. B. Nucleotide sequences of probes used for EMSA. PW60 contains three TTGAC sequences, which are mutated into TTGAA in mPW60. C. EMSA of binding of PW60 and mPW60 by recombinant WRKY18 protein (labelled as W18), WRKY40 protein (labelled as W40), and their mixture (labelled as W18+40). For each binding assay, 200 fmol recombinant proteins and 20 fmol labeled DNA probe were used.

Expression analysis using qRT-PCR showed that induction of *WRKY18 *and *WRKY40 *by ABA preceded that of *WRKY60 *(Figure 4A). Furthermore, ABA induction of *WRKY60 *was almost completely abolished in the *wrky18 *and *wrky40 *mutants (Figure 5C). These results suggest that *WRKY60 *might be directly regulated by WRKY18 and WRKY40. To examine this possibility, we compared the promoters of the three WRKY genes for presence of the TTGACC/T W boxes recognized by WRKY transcription factors. In the 1 kb promoter regions upstream of the coding sequences, there was a single WRKY box located at 240 bp upstream of the start codon of *WRKY18*. No TTGACC/T W box was found within the 1.0 kb upstream promoter sequence of *WRKY40*. Interestingly, there are three TTGACC/T W box sequences within a 19-bp region from position -791 to position -773 upstream of the translation start site of *WRKY60 *(Figure 6A). Presence of a cluster of W-boxes in the *WRKY60 *gene promoter suggests a possible role of WRKY proteins in the regulation of *WRKY60 *gene expression.

To determine whether the W boxes from the *WRKY60 *gene promoter are recognized by WRKY18 and WRKY40 proteins, we generated and labelled a double-stranded DNA probe containing these three W boxes (PW60) (Figure 6B). When incubated with recombinant WRKY18 or WRKY40 proteins, the probe produced a retarded band in electrophoretic mobility shift assays (Figure 6C). A similar retarded band was also produced when the probe was incubated with a mixture of WRKY18 and WRKY40 recombinant proteins (Figure 6C). To determine whether the W-boxes in the PW60 probe were important for the recognition, we also tested a mutant probe (mPW60) in which the TTGAC sequence of each W-box was changed to TTGAA (Figure 6B). As shown in Figure 6C, this mutant probe failed to detect retarded bands when incubated with WRKY18 or WRKY40 proteins. Thus, WRKY18 and WRKY40 proteins recognize the W-box sequences in the *WRKY60 *gene promoter.

### Activation of the WRKY60 Promoter by WRKY18 and WRKY40 in Protoplasts

**Figure 7:**
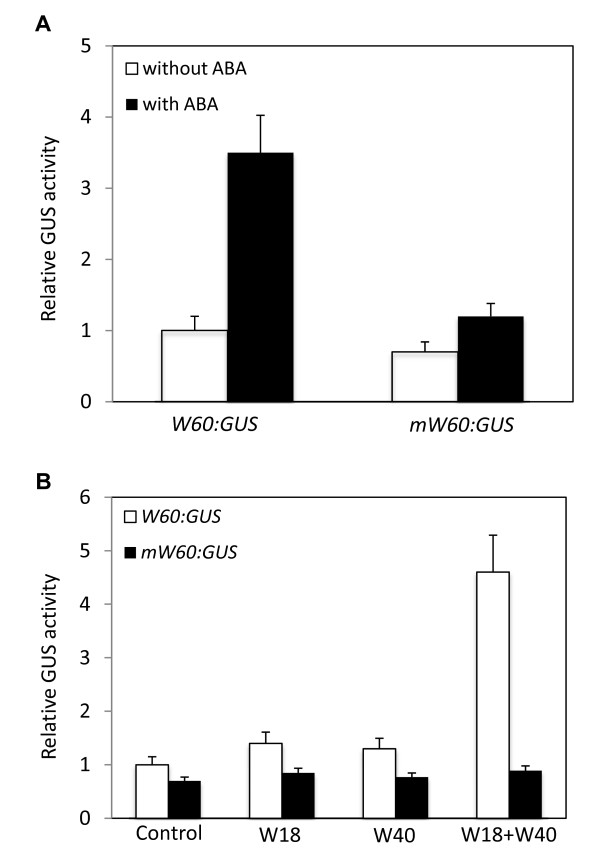
**Analysis of the *WRKY60:GUS *reporter gene using protoplast transfection**. A. Effects of ABA and W boxes on the *WRKY60 *promoter activity. Protoplasts from Col-0 wild type plants were transfected with the GUS reporter gene driven by the *WRKY60 *promoter (*W60:GUS*) or a mutant *WRKY60 *promoter in which the cluster of W-box sequences between -791 and -773 relative to the translation start codon were deleted (*mW60:GUS*). GUS activities were measured without or 12 h after the addition of 2 μM ABA. B. Effects of co-transfected WRKY18 and WRKY40 on the *WRKY60 *promoter activity. Protoplasts from *wrky18/wrky40 *double mutant plants were cotransfected with the *W60:GUS *or *mW60:GUS *reporter gene and an effect plasmid expressing WRKY18 (W18), or WRKY40 (W40) or two effector plasmids expressing the two WRKY proteins (W18+W40) driven by the *WRKY60 *promoter (*W60:GUS*). An empty effector plasmid was used as control. GUS activities were measured 12 h after co-transfection.

To determine whether the cluster of W box sequences are important for ABA-induced expression of *WRKY60*, we isolated a ~1,000 bp promoter fragment upstream of the translational start of *WRKY60 *and fused it to the GUS reporter gene (*W60:GUS*). A mutant WRKY60 promoter, in which the cluster of the W box sequences from position -791 to position -773 upstream of the translation start site of *WRKY60 *were deleted by overlapping PCR, was also fused to the GUS reporter gene (*mW60:GUS*). As shown in Figure 7A, addition of ABA into the protoplasts transfected with the *W60:GUS *construct resulted in about 3.5-fold induction of the reporter gene expression compared with the non-induced condition. On the other hand, addition of ABA into the protoplasts transfected with the mutant *mW60:GUS *construct resulted in less than 1.5-fold induction of the reporter gene expression compared with the non-induced condition. This result indicated that the W box sequences are critical for ABA-induced expression of *WRKY60*.

To determine whether WRKY18 and WRKY40 can activate the WRKY60 promoter in protoplasts, we generated the *WRKY18 *and *WRKY40 *effector constructs under control of the constitutive *CaMV 35S *promoter. As shown in Figure 7B, coexpression of WRKY18 or WRKY40 led to only a very small increase in the reporter gene expression from the *W60:GUS *construct in the *wrky18/wrky40 *mutant protoplasts (Figure 7B). On the other hand, coexpression of both WRKY18 and WRKY40 activated the the reporter gene expression the *W60:GUS *construct by almost 5-fold in the *wrky18/wrky40 *mutant protoplasts (Figure 7B). This activation of the WRKY60 promoter by coexpression of WRKY18 and WRKY40 was not observed from the *mW60:GUS *construct (Figure 7B). Thus, WRKY18 and WRKY40 cooperate in the activation of the *WRKY60 *gene expression mostly likely through recognition of the W box sequence in the *WRKY60 *gene promoter.

### Transcription-regulating activity of WRKY18, WRKY40 and WRKY60

**Figure 8:**
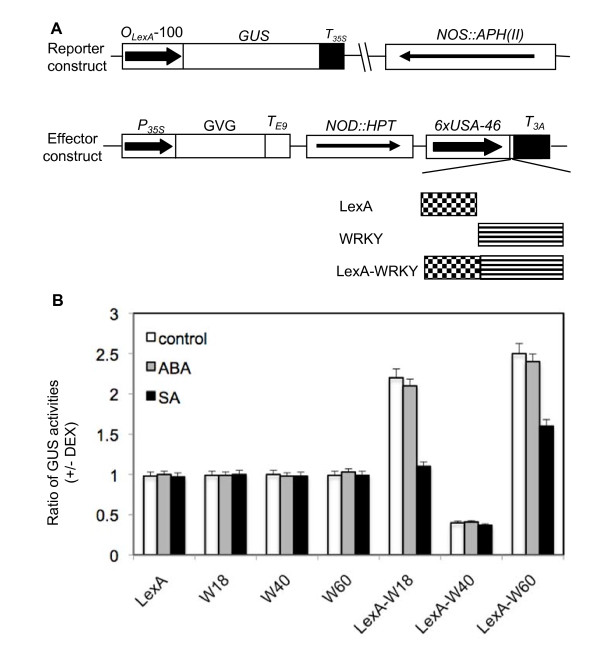
**The effect of ABA and SA on the transcription-regulating activities of WRKY18, WRKY40 and WRKY60**. A. Constructs of reporter and effector genes. The GUS reporter gene is driven by a synthetic promoter consisting of the -100 minimal CaMV 35S promoter and eight copies of the LexA operator sequence. The effector genes were cloned into pTA7002 behind the steroid-inducible promoter. The effector genes encode LexA DBD (LexA), WRKY and LexADBD-WRKY fusion protein, respectively. B. The effect of ABA and SA on the transcription-regulating activity of the WRKY proteins. Progeny from 5 independent transgenic lines for each effector gene were divided into three groups (15-20 plants/group) and sprayed with DEX (20 μM), DEX plus ABA (10 μM) or DEX plus SA (1 mM). Leaves were harvested at 0 and 24 hours after the treatment for assays of GUS activities and the ratios of GUS activities were calculated. Only those progeny that displayed induced expression of the effector genes as determined from RNA blotting following DEX treatment were used in the analyses. The means and errors were calculated from at least 15 positive progeny. The experiments were performed twice with similar results.

Functional analysis has revealed that structurally related and physically interacting WRKY18, WRKY40 and WRKY60 have a complex pattern of overlapping, antagonistic and distinct roles in plant defense and stress responses [27]. This complex pattern may, in part, result from the distinct transcriptional regulatory activities of the three transcription factors. To test this possibility, we employed a previously established transgenic system to determine the transcriptional regulatory activities of the three WRKY proteins through assays of a reporter gene in stably transformed plants [15]. The reporter gene in the system is a *GUS *gene driven by a synthetic promoter consisting of the -100 minimal *CaMV 35S *promoter and eight copies of the *LexA *operator sequence (Figure 8A). Because the minimal *35S *promoter is used, transgenic *Arabidopsis *plants harboring the reporter gene constitutively expressed only low levels of *GUS *and, therefore, it is possible to assay both transcription activation and repression by determining corresponding increase and decrease in GUS activities following co-expression of an effector protein.

To generate the WRKY18, WRKY40 and WRKY60 effectors, we fused their coding sequences with that of the DNA-binding domain (DBD) of LexA (Figure 8A). The fusion constructs were subcloned behind the steroid-inducible *Gal4 *promoter in pTA7002 [31] and transformed into transgenic plants that already contain the *GUS *reporter construct. Unfused *WRKY *and *LexA DBD *genes were also subcloned into pTA7002 and transformed into transgenic *GUS *reporter plants as controls (Figure 8A). For comparison, we also include WRKY48, a strong transcription activator [32], and WRKY7, a transcription repressor [15], in the assays. Transgenic plants containing both the reporter and an effector construct were identified through antibiotic resistance screens. To determine the effect of the effectors on *GUS *reporter gene expression, we determined the changes of GUS activities in the transgenic plants after induction of the effector gene expression by spraying 20 μM dexamethasone (DEX), a steroid. In the transgenic plants that expressed unfused WRKY18, WRKY40, WRKY60 or LexA DBD effector, there were little changes in the GUS activities after 18-hour DEX treatment (Additional file 3). In the transgenic plants harboring the *LexA DBD-WRKY18 *effector gene, induction of the fusion effector after DEX treatment resulted in 1.4 - fold increase in GUS activity (Additional file 3). A slightly higher 1.6-fold increase in GUS activity was observed in the transgenic plants harboring the *LexA DBD-WRKY60 *effector gene after DEX treatment (Additional file 3). By comparison, as previously reported [32], transgenic plants harboring the *LexA DBD-WRKY48 *effector gene, DEX treatment resulted in ~24-fold increase in GUS activity. These results indicate that both WRKY18 and WRKY60 are weak transcriptional activators. By contrast, in the transgenic plants harboring the *LexA DBD-WRKY40 *effector gene, induction of the fusion effector after DEX treatment resulted in a 2-fold reduction in GUS activity (Additional file 3). In transgenic plants harboring the *LexA DBD-WRKY7 *effector gene, DEX treatment resulted in ~5-fold reduction in GUS activity. Thus, WRKY40 is a relatively weak transcriptional repressor.

We have previously shown that WRKY18, WRKY40 and WRKY60 physically interact with themselves and with each other to form both homo- and hetero-complexes [27]. In addition, the three WRKY genes are induced by pathogen infection, SA and ABA treatment [27] (Figure 5). Thus, the transcription-regulating activity of the three WRKY proteins may change upon interaction with each other or with other induced proteins. To test this possibility, we examined the effects of SA and ABA treatment on the changes of GUS activities in the progeny of the transgenic effector/reporter lines after 24-hour DEX induction of the effector genes. Extension of DEX treatment from 18 to 24 hours increased significantly the expression levels the effector genes (unpublished data). In the transgenic plants that expressed unfused WRKY18, WRKY40, WRKY60 or LexA DBD effector, there were little changes in the GUS activities after DEX treatment with or without ABA or SA treatment (Figure 8B). In the transgenic plants harboring the *LexA DBD-WRKY18 *effector gene, induction of the fusion effector after DEX treatment resulted in 2.2 -fold increase in GUS activity (Figure 8B). ABA treatment had little effect on DEX-induced change of GUS activity, suggesting that ABA did not significantly affect the transcription-activating activity of WRKY18. On the other hand, in SA-treated transgenic plants harboring the *LexA DBD-WRKY18 *effector gene, there was almost no increase in GUS activity following induction of the fusion effector after DEX treatment. Thus, SA treatment almost completely abolished the transcription-activating activity of WRKY18. In the absence of ABA or SA treatment, a 2.5-fold increase in GUS activity was observed in the transgenic plants harboring the *LexA DBD-WRKY60 *effector gene after 24-hour DEX treatment (Figure 8B). Again ABA treatment had little effect on DEX-induced change of GUS activity while SA treatment resulted in more than 50% reduction in the increase of GUS activity following 24-hour DEX induction of the fused *LexA DBD-WRKY60 *effector gene (Figure 8B). In the transgenic plants harboring the *LexA DBD-WRKY40 *effector gene, induction of the fusion effector after DEX treatment resulted in a 2.5-fold reduction in GUS activity (Figure 8B). Neither ABA nor SA treatment had significant effect on the change of GUS activities in the transgenic plants harboring the *LexA DBD-WRKY40 *effector gene (Figure 8B). Thus, the transcription-regulating activity of both WRKY18 and WRKY60, but not WRKY40, was substantially altered by SA treatment.

### Expression of ABA related genes

**Figure 9:**
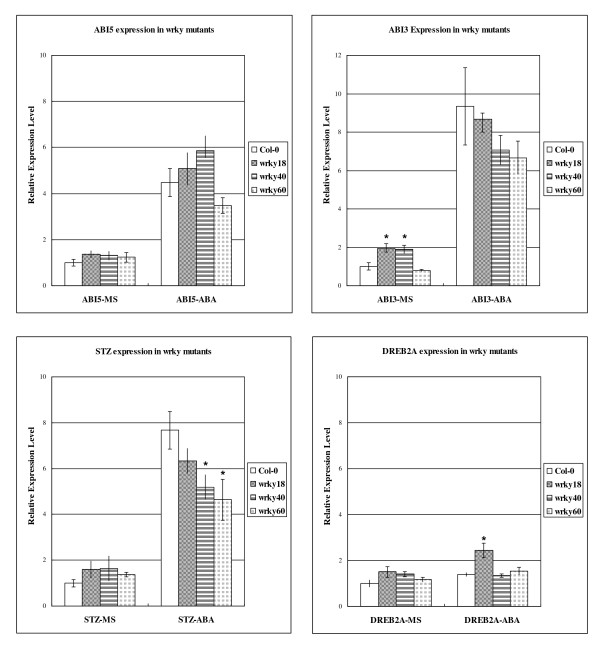
**RNA levels of *ABI3*, *ABI5*, *DREB2A *and *STZ *in *wrky18, 40, 60 *mutants and wild type seedlings**. Seedlings of wild type or mutants were grown on MS medium for 14 days before being transplanted onto MS plates with or without 2.0 μM ABA. RNA was extracted from seedlings on MS medium 12 hours after transplantation. Relative RNA levels of the 4 genes ABI3, ABI5, DREB2A and STZ were analyzed using gene-specific primers by real-time PCR. The means and standard errors were calculated from three independent experiments, all of which included no less than 20 seedlings per sample. Asterisks mark statistically significant differences of expression level between genotypically identical seedlings with or without ABA treatment, by Student-Newman-Keuls Test(p-value < 0.05).

To further understand how the three WRKY proteins are involved in the regulation of ABA responses, we compared wild type and the mutants for the three WRKY mutants for expression of four genes associated with ABA signalling; *ABI5*, *ABI3*, *STZ *and *DREB2A*. As shown in Figure 9 for *ABI5*, *STZ *and *DREB2A*, we observed no significant difference between the wild type and the mutants when the seedlings were grown in ABA-less MS grown medium. For *ABI3*, the basal level were slightly but significantly higher in the *wrky18 *and *wrky40 *mutant plants(Figure 9). On the ABA-containing medium, we observed modest but significant reduction in expression of *STZ *in the *wrky60 *mutant (Figure 9). There was also relatively small reduction in and *STZ *expression in the *wrky40 *mutant. Surprisingly, no significant reduction of the ABA-related genes was observed in the *wrky18 *mutant; in fact, there appear to be a small but significant increase in ABA-induced expression of *DREB2A *in the *wrky18 *mutant when compared to wild type (Figure 9).

## Discussion

### Differential roles of WRKY18, WRKY40 and WRKY60 in ABA and abiotic stress responses

Over the last several years, there has been growing evidence that plant WRKY transcription factors are involved in plant ABA signaling and abiotic stress responses. In rice and barley, ABA induces expression of a number of WRKY genes in aleurone cells [23,24,33,34]. When transiently overexpressed in aleurone cells, some of these ABA-inducible WRKY genes activate or repress ABA-inducible reporter genes. A number of studies have also shown that WRKY genes are induced by a variety of abiotic stress conditions and overexpression of some WRKY genes altered plant stress tolerance. In the present study, we have determined the role of three Arabidopsis WRKY genes in plant ABA signaling by analyzing the effects of ABA on germination, root growth of their knockout mutants and overexpression lines. We have demonstrated that while disruption of *WRKY18 *and *WRKY60 *caused reduced sensitivity to ABA, disruption of *WRKY40 *increased ABA sensitivity for inhibition of germination and root growth (Figures 1 and 2). Likewise, we have demonstrated that the *wrky18 *and *wrky60 *mutants but not the *wrky40 *mutant are more tolerant to salt and osmotic stress (Figure 3). The differential roles of the three structurally related WRKY proteins in plant ABA and abiotic stress responses were also demonstrated from the analysis of the double and triple knockout mutants and overexpression lines (Figure 1, 2 and 3).

**Figure 10:**
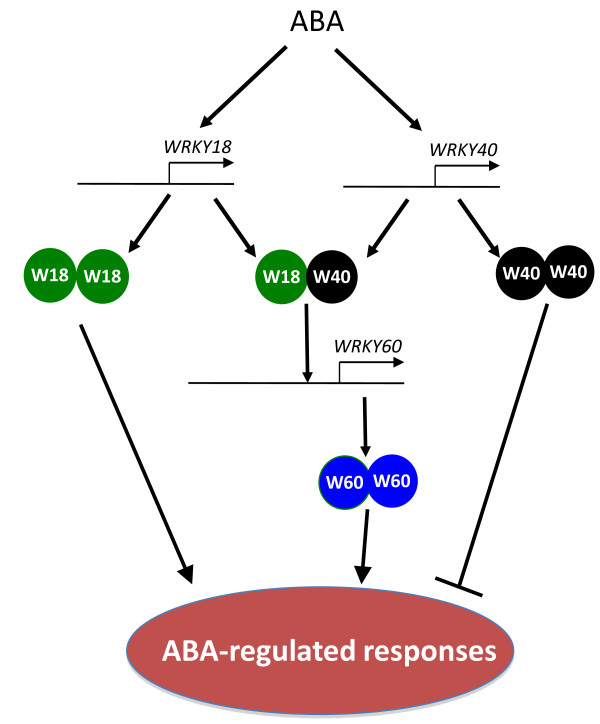
**Proposed model for involvement of WRKY18, WRKY40 and WRKY60 in ABA responses**. ABA induction of WRKY18 and WRKY40 leads to increase in WRKY18 and WRKY40 proteins that form both homo- and heterocomplexes through physical interactions. The requirement of both WRKY18 and WRKY40 for induction of *WRKY60 *suggest possible involvement of a WRKY18/WRKY40 heterocomplex that may recognize the W box sequences in the WRKY60 gene promoter and activate its expression. WRKY18 and WRKY60 positively regulate while WRKY40 negatively regulates plant responses to ABA probably by modulating ABA-regulated genes.

The role of ABA during seed germination has been extensively studied. The opposite phenotypes of the *wrky *mutants in ABA sensitivity for inhibition of germination strongly suggest that these WRKY genes function as either positive or negative regulators of ABA signaling. Although no altered phenotypes of the *wrky40 *mutant was observed in ABA effects on root growth or salt and osmotic sensitivity, which could be due to low sensitivity of the assays, we did observe that the *wrky18 *and *wrky60 *mutants exhibited reduced ABA inhibition of root growth as well as reduced sensitivity to salt and osmotic stress (Figure 1, 2 and 3). Therefore, it is possible that altered phenotypes in abiotic stress are related to altered ABA signaling in the *WRKY *gene mutants. For example, the higher level of *DREB2A *in *wrky18 *mutant than in wild type plants under exogenous ABA treatment may partially explain the higher abiotic resistanc(Figure 1,2, 3 and 9), considering overexpression of transcriptional activation domain of DREB2A resulted in significant drought stress tolerance[35]. It is known that the inhibited effect of ABA on root growth involves pathways mediated by other plant hormones such as ethylene, auxin and jasmonic acid. The relationship between ABA signaling and salt and osmotic stress tolerance is also very complex. In some mutants such as tomato *tss2 *mutant, ABA hypersensitivity is associated with osmotic stress hypersensitivity [36,37]. In other mutants such as the *tos *mutant, ABA insensitivity is associated with osmotic stress hypersensitivity [38]. These studies suggest that proper levels of ABA perception and signaling are important for the abiotic stress tolerance. WRKY18 and WRKY60 are weak transcriptional activators and WRKY40 is a weak transcriptional repressor (Figure 8). The relatively weak transcription regulatory activities would make the three transcription factors suitable as either positive or negative regulators for modulating ABA signaling and influencing ABA-regulated plant growth and abiotic stress responses (Figure 10).

The roles of WRKY18, WRKY40 and WRKY60 in ABA signaling are consistent with the ABA-inducible expression of the three genes (Figure 4). Interestingly, the three WRKY genes display distinct expression patterns upon ABA treatment. *WRKY18 *and *WRKY40 *are rapidly induced upon ABA treatment and are required for ABA-induced *WRKY60 *expression (Figure 4). On the other hand, ABA-induced expression of *WRKY60 *is delayed but also prolonged (Figure 4). In addition, *WRKY60 *and *WRKY40 *act partially redundantly in repressing *WRKY18 *expression (Figure 5). This expression pattern raises the possibility that the three WRKY proteins are part of a regulatory network that modulates gene expression in the ABA signaling pathway. Upon ABA induction, *WRKY18 *and *WRKY40 *are first induced and their products could act as early transcriptional effectors to regulate expression of additional ABA-induced genes including *WRKY60 *(Figure 10). Induced WRKY60 would then act with WRKY40 to repress *WRKY18*, forming a negative feedback loop. The prolonged expression and the transcription-activating activity of WRKY60 would allow it to have a relatively sustained effect on ABA-regulated gene expression. This interpretation is consistent with the relatively strong phenotypes of the *wrky60 *mutant in ABA and stress tolerance when compared to those of the *wrky18 *mutant (Figure 1, 2 and 3).

### Roles of WRKY18, WRKY40 and WRKY60 in crosstalk between abiotic and biotic responses

We have previously shown that single *wrky18*, *wrky40 *and *wrky60 *mutants exhibited no or small alterations in response to the hemibiotrophic bacterial pathogen *P. syringae *or the necrotrophic fungal pathogen *B. cinerea *[27]. However, *wrky18 wrky40 *and *wrky18 wrky60 *double mutants and the *wrky18 wrky40 wrky60 *triple mutant were substantially more resistant to *P. syringae *but more susceptible to *B. cinerea *than wild-type plants [27]. These phenotypes and additional analysis of SA- and JA-regulated gene expression suggest that these WRKY proteins have a partially redundant negative effect on SA-mediated defense but exerted a positive role in JA-mediated defense. Likewise, we have shown in this report that WRKY18 and WRKY60 positively regulate while WRKY40 negatively regulates plant ABA response (Figure 1, 2 and 3). As ABA is known to counteract SA-defense [7] but function as a signal in JA-mediated defense against necrotrophic pathogens [28], the roles of these three WRKY proteins in plant defense and ABA and stress responses might be mechanistically linked. This notion is particularly attractive for WRKY18 and WRKY60, which might negatively impact SA-dependent defense through positively modulating ABA signaling. On the other hand, WRKY40 antagonized WRKY18 and WRKY60 in ABA response but functions partially redundantly with WRKY18 and WRKY60 in SA-dependent defense. As will be discussed later, WRKY18, WRKY40 and WRKY60 interact with themselves and with each other to form distinct complexes that may differ in both DNA-binding and transcription-regulating activities. The interacting partners of WRKY40 formed during pathogen infection might not be the same as those in ABA-treated plants and, therefore, may function in distinct manners during plant defense and stress responses.

### Molecular basis of functional interactions among WRKY18, WRKY40 and WRKY60

We have previously shown that through a leucine-zipper motif present at the N-terminus of the three proteins, WRKY18, WRKY40 and WRKY60 interacts with themselves and with each other to form both homo-complexes and hetero-complexes with altered DNA binding activities [27]. In the present study, we have shown that WRKY18 and WRKY60 act as weak transcriptional activators and WRKY40 is a transcriptional repressor in plant cells (Figure 8). Furthermore, we have shown that SA treatment can diminish or reduce the transcription-activating activity of WRKY18 and WRKY60 (Figure 8). Thus, the three WRKY proteins may form a range of protein complexes with distinct DNA-binding and transcription-activating or -repressing activities. The complex pattern of DNA binding and transcription regulatory activities of the three WRKY proteins may explain their complex biological roles in plant defense and stress responses.

In plant defense responses, analysis of T-DNA insertion mutants indicated that WRKY18, WRKY40 and WRKY60 have redundant repressor function in plant defense against virulent hemibiotrophic *P. syringae *and biotrophic *Golovinomyces orontii *[27,39]. Genome-wide gene expression profiling experiments also showed that WRKY18 and WRKY40 have a redundant role in repressing a subset of 23 genes associated with PAMP-triggered immunity [39]. The redundant roles of WRKY18 and WRKY40 as repressors of plant defense genes are consistent with the demonstrated repressing activity of WRKY40 but not with the transcription-activating activity of WRKY18. However, we have also shown that after treatment with SA, which is elevated in pathogen-infected plants, the transcription-activating activity of WRKY18 is largely diminished (Figure 8). Under such conditions WRKY18 may compete for binding to promoter sequences with other pathogen-induced WRKY proteins with stronger transcription-activating activities, thereby preventing strong expression of the target genes. In the absence of SA treatment or pathogen infection, on the other hand, WRKY18 may function as a positive regulator of plant disease resistance by acting as an activator of plant defense genes as observed in transgenic *WRKY18*-overexpressing plants [40]. The positive role of WRKY18 as a positive regulator of disease resistance and activator of defense gene would be antagonized by the transcription-repressing WRKY40 if they are co-exppressed. Indeed, we have previously observed that potentiated defense responses in *WRKY18*-overexpressing Arabidopsis plants are abolished by co-overexpression of *WRKY40 *in the same transgenic plants [40].

The differential roles of the three WRKY proteins in plant responses to ABA and abiotic stress conditions are correlated with their distinct transcriptional regulatory activities. WRKY18 and WRKY60 act as transcriptional activators and functional as positive regulators of plant ABA and abiotic stress responses. By contrast, WRKY40 acts as a transcriptional repressor and functional as a negative regulator of plant ABA responses. Thus, it is mostly likely that the roles of the three WRKY proteins in plant ABA and stress responses are mediated by their activities in activating or repressing plant genes involved in ABA and stress signaling.

ABA-induced expression of *WRKY60 *is severely compromised in both the *wrky18 *and *wrky40 *single mutants (Figure 6C). Thus, both WRKY18 and WRKY40 are important for ABA-induced *WRKY60 *expression. In the promoter of *WRKY60*, there is a cluster of three W boxes within a 19 bp region (Figure 6A), which are important for ABA-induced expression of WRKY60 in protoplasts (Figure 7A). Using EMSA, we have shown that the cluster of W boxes in the *WRKY60 *gene promoter is recognized by both WRKY18 and WRKY40 (Figure 6C). Protoplast transfection assays further showed that only co-overexpression of WRKY18 and WRKY40 but not WRKY18 or WRKY40 alone led to activation of the WRKY60 gene promoter and this activation of WRKY60 was dependent on the cluster of three W boxes in its promoter (Figure 7). It is possible that upon ABA treatment, WRKY18 and WRKY40 are first induced and cooperative binding of induced WRKY18 and WRKY40 or binding of a WRKY18/WRKY40 heterocomplex to the cluster of W boxes in the *WRKY60 *promoter is necessary for the subsequent induction of *WRKY60 *(Figure 10).

## Conclusions

We have found that mutants and overexpression lines for Arabidopsis *WRKY18*, *WRKY40 *and *WRKY60 *genes have altered phenotypes in plant sensitivity to ABA, salt and osmotic stress. Thus, the three WRKY transcription factors play roles in both plant biotic and abiotic stress responses. Additional studies of their expression, DNA binding and transcription-regulating activities strongly suggest that the three WRKY transcription factors form a highly interacting regulatory network that modulates gene expression in both plant defense and stress responses.

## Methods

### Materials and Growth Conditions

The Arabidopsis knockout mutants and overexpression lines for *WRKY18*, *WRKY40 *and *WRKY60 *have been previously described[27]. The plants were grown in mixture of peat/forest soil (purchased from Pingstrup Substrate) and vermiculite (3:1) in a green house at 23°C with 150 μE m^-2 ^s^-1 ^light on a photoperiod of 12 h light and 12 h dark.

### Assays of Sensitivity to ABA and Stress

Seeds (100 seeds for each replicate) of wild type, mutants and overexpression lines were surface sterilized by treating for 5 min in 15% bleach and 0.5% Tween-20. The sterilized seeds were placed on 1/2 Murashige and Skoog medium (Gibcol) and 0.3% phytagel (Sigma) and stratified at 4°C for 4 days before transfer to 23°C for germination and growth. For tests of the ABA effect on germination, seeds were plated directly onto media containing various concentrations of ABA. For testing root elongation under ABA or abiotic stress treatments, seeds were firstly germinated on MS media. Four-days-old seedlings were then transferred to media containing ABA, mannitol, PEG or NaCl. Root length was measured 7 days after transfer using the NIH ImageJ1.41 program.

### Cloning, expression, purification of recombinant proteins and the EMSA

Cloning, expression in *E. coli *and purification of recombinant WRKY 18 and 40 proteins have been previously described [27]. 5' biotin labeled DNA probes of the *WRKY60 *promoter was synthesized by Invitrogen. EMSA and detection were performed according to the manual of the Pierce's Lightshift Chemiluminescent EMSA Kit. In each binding assay, 200 fmol recombinant WRKY protein and 20 fmol DNA probe were used.

### Gene expression analysis

Total RNA was extracted from plant samples following the instructions in handbook of Trizol (Invitrogen) and treated with RNase-free DNase I (Promega) to remove contaminated DNA. cDNA was synthesized by adding 100 ng total RNA into 10 μl reaction with random hexames and oligo dT primers provided by PrimeScript RT Reagent Kit (Takara). Quantitative-real time PCR was performed in ABI7900 HT machine with SYBR PrimeScript RT-PCR Kit (Takara). The RT-reaction product (2 μl) was used as template in a 25 μl PCR mixture. The following program was used for PCR amplification: Initial denaturation at 95°C 10 sec. followed by 40 cycles of 95°C 5 sec. and 60°C 30 sec. The β-actin gene was used as endogenous reference gene. Data analysis was performed using the ABI SDS 2.0 program. The primers used in real time PCR are listed in Additional file 4.

### Protoplast transfection assays

The full-length GUS gene was clone into the XbaI site of pFF19 [41]. The 1.0 kb WRKY60 promoter was PCR-amplified using the following two primers: atgcaagcTTTCTTTGTTTTCTGCCGGTTT and atgcgagctcAAATTTAGGTTCACAGGAGCCA. The amplified promoter DNA was digested with HindIII and SacI and was used to replace the *CaMV 35S *promoter in pFF19. The mutant *WRKY60 *promoter in which the cluster of W-box sequences between -791 and -773 relative to the translation start codon was generated by overlapping PCR. The sequences of the promoters were verified by DNA sequencing.

To generate the *WRKY18 and WRKY40 *effector constructs, their cDNA fragments that contained the full coding sequences and the 3'-untranslated regions were excised from their respective cloning plasmids and subcloned into the same restriction sites of pFF19 in the sense orientation behind the *35S *promoter.

Protoplast isolation and transfection were carried out according to the protocols as previously described [42]. Four- to five-weeks old rosette leaves were used for isolation of mesophyll protoplasts. Protoplast transfection was performed using 40% polyethylene glycol with 10 μg reporter plasmid and 15 μg effector plasmid DNA.

### Assays of Transcriptional Regulatory Activity

Transgenic *Arabidopsis *plants containing a *GUS *reporter gene driven by a synthetic promoter consisting of the -100 minimal *CaMV 35S *promoter and eight copies of the *LexA *operator sequence were previously described [15]. To generate effector genes, the DNA fragment for the LexA DBD was digested from the plasmid pEG202 (Clontech) using *Hind*III and *EcoR*I and cloned into the same sites in pBluescript. The full-length *WRKY18, WRKY40 *and *WRKY60 *cDNA fragments were subsequently subcloned behind the *LexA DBD *to generate translational fusions. The *LexA DBD-WRKY *fusion genes were cloned into the *Xho*I and *Spe*I site of pTA2002 behind the steroid-inducible promoter [31]. As controls, the unfused *LexADBD *and *WRKY *genes were also cloned into the same sites of PTA7002. These effector constructs were directly transformed into the transgenic *GUS *reporter plants and double transformants were identified through screening for antibiotic (hygromycin) resistance. Determination of activation or repression of *GUS *reporter gene expression by the effector proteins was performed as previously described [15]. For determining the effect of ABA and SA on the transcription-regulating activity of the WRKY proteins, progeny from 5 independent transgenic lines for each effector gene were divided into three groups (15-20 plants/group) and sprayed with DEX (20 μM), DEX plus ABA (10 μM) or DEX plus SA (1 mM). Leaves were harvested at 0 and 24 hours after the treatment for assays of GUS activities.

## Authors' contributions

HC performed major part of phenotype assays, expression assay and EMSA assay and composed the draft. LZ analyzed the activator/repressor activity of the WRKY proteins. JS composed the draft with HC and help in studying the phenotypes. YX helped analyzing expression patterns. ZC participated in the design of the study and edited the manuscript. XX conceived of the study, participated in the design of the study and edited the manuscript. All authors read and approved the final manuscript.

## Supplementary Material

Additional file 1 **Altered germination rates under exogenous ABA treatment**. Seeds of wild type, mutants and overexpression lines were sown on 1/2 MS media containing indicated concentrations of ABA. Seedlings with green cotyledons were considered as germinated. Click here for file

Additional file 2 **Altered stress tolerance of the WRKY mutants**. Seeds of wild type and mutants were grown on 1/2 MS media for four days and then were transferred to MS agar media without or with -0.75 MPa PEG, 200 mM mannitol or 150 mM NaCl. The picture was taken and the root length was determined at the 7th day after the transfer. The average root length of each genotype in MS medium and their standard errors were calculated from three independent experiments, every of each employed more than 25 seedlings per genotype. Relative root length was the ratio of average root lengths of seedlings in medium with -0.75 MPa PEG, 200 mM mannitol or 150 mM NaCl to those in MS medium. Click here for file

Additional file 3 **Transcription-regulating activities of WRKY18, WRKY40 and WRKY60**. The ratios of GUS activities were calculated from the GUS activities determined in the leaves harvested 18 hours after DEX treatment (+) over those determined prior to DEX treatment (-). Only those transformants that displayed induced expression of the effector genes as determined from RNA blotting following DEX treatment were used in the analyses. The means and errors were calculated from at least 15 positive transformants. The experiments were performed twice with similar results. Click here for file

Additional file 4 **Primer sequences for qRT-PCR assay**. The designs of these primers were based on mRNA sequence of from At4g31800, At1g80840, At2g25000, AT5G05410.1, AT1G27730, AT2G36270 and AT3G24650 respectively and generated single sharp peeks in melt curves. Click here for file
