# Supplementary figures and images for: Roles of arabidopsis WRKY18, WRKY40 and WRKY60 transcription factors in plant responses to abscisic acid and abiotic stress
Source: BMC Plant Biol. 2010 Dec 19;10:281. doi: 10.1186/1471-2229-10-281 (PMC3023790; doi:10.1186/1471-2229-10-281)

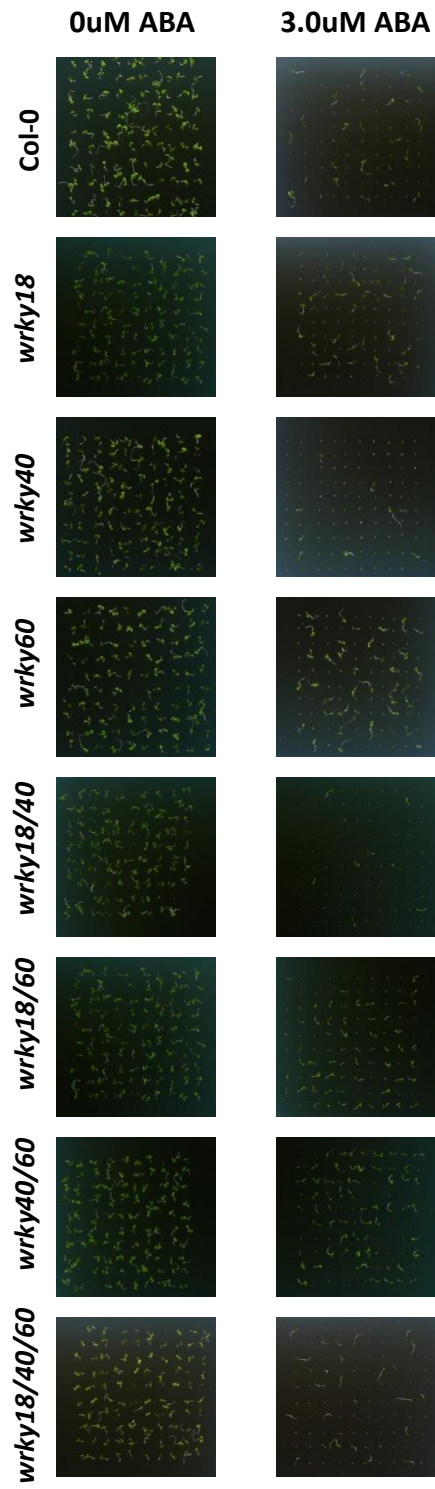

Supplement: Additional file 1 — Altered germination rates under exogenous ABA treatment. Seeds of wild type, mutants and overexpression lines were sown on 1/2 MS media containing indicated concentrations of ABA. Seedlings with green cotyledons were considered as germinated. [file 1471-2229-10-281-S1.PDF]

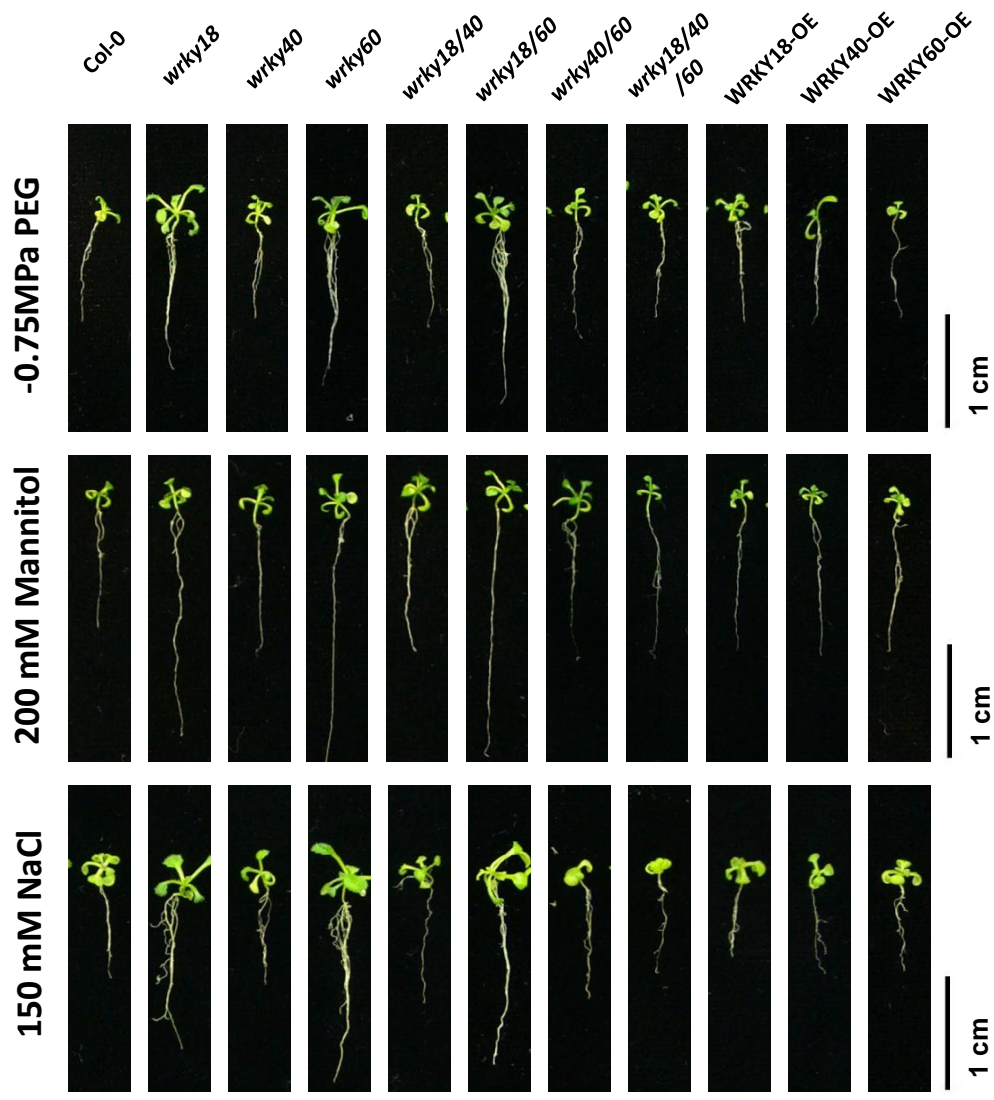

Supplement: Additional file 2 — Altered stress tolerance of the WRKY mutants. Seeds of wild type and mutants were grown on 1/2 MS media for four days and then were transferred to MS agar media without or with -0.75 MPa PEG, 200 mM mannitol or 150 mM NaCl. The picture was taken and the root length was determined at the 7th day after the transfer. The average root length of each genotype in MS medium and their standard errors were calculated from three independent experiments, every of each employed more than 25 seedlings per genotype. Relative root length was the ratio of average root lengths of seedlings in medium with -0.75 MPa PEG, 200 mM mannitol or 150 mM NaCl to those in MS medium. [file 1471-2229-10-281-S2.PDF]

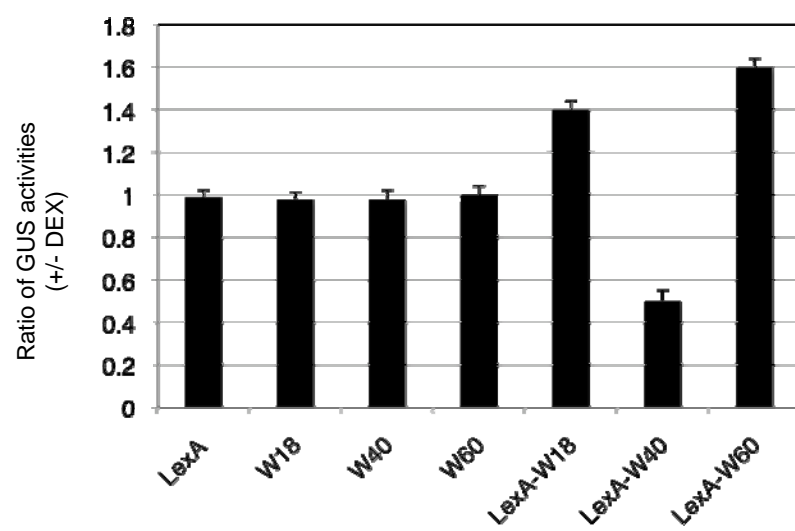

Supplement: Additional file 3 — Transcription-regulating activities of WRKY18, WRKY40 and WRKY60. The ratios of GUS activities were calculated from the GUS activities determined in the leaves harvested 18 hours after DEX treatment (+) over those determined prior to DEX treatment (-). Only those transformants that displayed induced expression of the effector genes as determined from RNA blotting following DEX treatment were used in the analyses. The means and errors were calculated from at least 15 positive transformants. The experiments were performed twice with similar results. [file 1471-2229-10-281-S3.PDF]
