# Supplementary material for: Roles of arabidopsis WRKY18, WRKY40 and WRKY60 transcription factors in plant responses to abscisic acid and abiotic stress
Source: BMC Plant Biol. 2010 Dec 19;10:281. doi: 10.1186/1471-2229-10-281 (PMC3023790; doi:10.1186/1471-2229-10-281)
Supplement: Additional file 4 — Primer sequences for qRT-PCR assay. The designs of these primers were based on mRNA sequence of from At4g31800, At1g80840, At2g25000, AT5G05410.1, AT1G27730, AT2G36270 and AT3G24650 respectively and generated single sharp peeks in melt curves. [file 1471-2229-10-281-S4.PDF]

Primer	Sequence
AtWRKY18 real time PCR primer-s	5' CGTGCCTACTGAAACATCGGAC 3'
AtWRKY18 real time PCR primer-a	5' GTAAGCTCTAGGTGACGGGTTGTC 3'
AtWRKY40 real time PCR primer-s	5' AAATCAGCCCTCCCAAGAAACG 3'
AtWRKY40 real time PCR primer-a	5' CTTCACGACAGTCTCTTCTCTCTGC 3'
AtWRKY60 real time PCR primer-s	5' GGTGGGCTTGAACCAGTTGAGG 3'
AtWRKY60 real time PCR primer-a	5' AATCTCCCGGAAATAGCAGTCG 3'
β-actin real time PCR primer-s	5' GGTAACATTGTGCTCAGTGGTGG 3'
β-actin real time PCR primer-a	5' AACGACCTTAATCTTCATGCTGC 3'
DREB2A real time PCR primer-s	5' TCTGGGAAGGAGATGGCAGT 3’
DREB2A real time PCR primer-a	5' AGCCACAGTAGTACCGTCACCTC 3’
STZ/ZAT10 real time PCR primer-s	5' AGGCTCTTACATCACCAAGATTAG 3’
STZ/ZAT10 real time PCR primer-a	5' TACACTTGTAGCTCAACTTCTCCA 3’
ABI3 real time PCR primer-s	5' ACTGTGACGACTCTTCTGGTGCAT 3’
ABI3 real time PCR primer-a	5' ATCAATGCAATCACCACCGCCTTC 3’
ABI5 real time PCR primer-s	5' GAGAATGCGCAGCTAAAACA 3’
ABI5 real time PCR primer-a	5' GTGGACAACTCGGGTTCCTC 3’
